# A Comparative In Vitro Physicochemical Analysis of Resin Infiltrants Doped With Bioactive Glasses

**DOI:** 10.7759/cureus.64500

**Published:** 2024-07-13

**Authors:** Syed Zubairuddin Ahmed, Abdul S Khan, Naemah M Aljeshi, Budi A Md Sabri, Sultan Akhtar, Mohamed Ibrahim Abu Hassan

**Affiliations:** 1 Restorative Dental Sciences, College of Dentistry, Imam Abdulrahman Bin Faisal University, Dammam, SAU; 2 Dentistry, College of Dentistry, Imam Abdulrahman Bin Faisal University, Dammam, SAU; 3 Dentistry, College of Dentistry, University Teknologi Mara, Sungai Buloh, MYS; 4 Department of Biophysics, Institute for Research and Medical Consultations, Imam Abdulrahman Bin Faisal University, Dammam, SAU; 5 Restorative Dental Sciences, College of Dentistry, MAHSA University, Slengor, MYS

**Keywords:** surface roughness and microhardness, scanning electron microscopy, ph cycling, artificial saliva, resin infiltrants

## Abstract

Objective

This study aimed to investigate the longevity and effectiveness of bioactive glass (BAG)-based dental resin infiltrants.

Materials and methods

The three types of BAG - 45S5 bioglass (RIS), boron-substituted (RIB), and fluoride-substituted (RIF) - were incorporated with photoinitiated dimethacrylate monomers to create experimental resin infiltrants. ICON^®^ (CN; DMG-America, Ridgefield Park, NJ) and pure resin (PR) were used as control groups in this study. Disc-shaped samples were prepared for the experimental and control groups. The samples were challenged with the pH cycle and immersed in the artificial saliva for 30 days. On Day 0 and Day 30, the pH cycle and artificial saliva immersion, Vicker’s microhardness, surface roughness, and surface morphology were investigated.

Results

The RIF group's disc samples showed the highest Vicker’s microhardness values (78.20 ±0.06) on Day 30 of artificial saliva immersion, whereas the CN group's values were the lowest (55.99 ±0.24). Following the pH cycling, the RIF displayed the highest hardness (64.15 ±1.89) whereas the CN group's values were the lowest (33.47 ±1.28). Regarding surface roughness, on Day 30, the RIB resin group exhibited the highest (1.14 ±0.001 µm). In contrast, the CN resin showed the lowest (1.07 ±0.06 µm) values, while immersed in the artificial saliva solution. In the same duration of time, in the pH cycling solution, PR showed the least (0.85 ±0.89 µm), while RIF showed the highest roughness value (0.94 ±0.54 µm). Morphological analysis revealed that following the artificial saliva immersion, the RIB, CN, and PR exhibited smoother surfaces compared to the RIS and RIF groups. However, when immersed in the pH cycling solution, RIB and RIF showed more resistance against acid attack.

Conclusions

Our results revealed that the experimental resin groups performed much better than the commercial resin infiltrants following artificial saliva and pH cycling challenges.

## Introduction

Tooth decay, also referred to as dental caries, is one of the most common chronic disorders worldwide, affecting people of all ages [1]. Caries remain a major public health concern, leading to discomfort, tooth loss, and a lower quality of life, even with significant improvements in dental care and preventive measures [2]. An estimated two billion people suffer from caries of permanent teeth, and 514 million children suffer from caries of primary teeth worldwide [3]. The World Health Organization (WHO) reports that most adults and approximately 60-90% of school-aged children suffer from dental caries [4]. According to the 2019 Global Burden of Disease Study, tooth decay that goes untreated in permanent teeth is the most common ailment, impacting around 2.3 billion people worldwide [5]. White spot lesions (WSLs) are considered initial caries or enamel caries that first develop as opaque, chalky white patches on the surface of the tooth due to subsurface demineralization. These lesions pose a serious clinical problem, especially in orthodontic patients where the risk of demineralization is increased by plaque buildup around brackets and bands [6].

There has been a paradigm shift in the past few years in managing dental caries lesions. These changes have played a major role in the evolution of the conventional concept of treating dental caries. Fluoride therapy, resin infiltration, remineralizing agents, and esthetic restorative materials have all been assessed for efficacy, benefits, and limitations [7]. Whether initial or complex, the current approach to managing carious lesions emphasizes prevention through non-invasive methods or minimal intervention. This includes measures like oral hygiene practices (such as flossing and interdental brushes) and fluoride application to reduce the risk of caries progression [8].

Resin infiltrants are advanced materials that are less viscous, cured with light, and can quickly flow into demineralized tooth structures through capillary action [9]. These infiltrants prevent the enamel lesion progression and provide a high-end conservative approach. Despite some limitations (microhardness and color stability), these infiltrants are highly capable of deferring the expected invasive treatment approach to dealing with these WSLs [10]. Existing resin infiltrants are characterized by their physicochemical properties, including low viscosity, light-curability, and the ability to penetrate demineralized enamel through capillary action. The primary purpose of resin infiltrants is to arrest the progression of carious lesions and improve the esthetic appearance of affected teeth without requiring invasive procedures [11]. However, these materials have certain shortcomings, including their inability to induce significant enamel remineralization. The application process can also be technique-sensitive, requiring precise isolation and moisture control [12].

Recent advancements in dental materials have witnessed the emergence of bioactive glass (BAG)-based products, which appear as a promising alternative for remineralization [13]. BAG is composed primarily of silica (SiO_2_), calcium oxide (CaO), sodium oxide (Na_2_O), and phosphorus pentoxide (P_2_O_5_), and has demonstrated excellent biocompatibility and the ability to bond with biological tissues [14]. BAG-based resin infiltrants represent a novel class of dental materials designed for the minimally invasive treatment of early carious lesions by combining the caries-inhibiting properties of BAG with the stabilizing effects of resin [15]. When used as part of a resin infiltrant, these glasses can halt the progression of caries by creating a barrier [16].

BAG can release calcium and phosphate ions, which can potentially remineralize WSLs [17]. Several studies have combined resin infiltrants with BAG to make it an excellent material that not only preserves the tooth structure in its initial stages [18] but also enhances its chemical properties and hence overcomes shortcomings (color stability, ion leaching capability, and aging) [10]. Physicochemical analyses of these materials are crucial as they provide insights into the chemical interactions between the infiltrant and tooth structure in the oral environment [18]. WSL surface characteristics seem to be enhanced by resin infiltration in conjunction with BAG remineralization or CPP-ACPF remineralization [19]. However, very few studies have used different types of BAG, while most of the researchers have used BAG along with other combinations of treating WSLs, to enhance its efficacy.

In this study, three distinct forms of BAG - (1) 45S5 bioglass (RIS), (2) boron-doped BAG (RIB), and (3) fluoride-doped BAG (RIF) - were incorporated into the dental resin to investigate the physical and chemical properties compared to the commercial material. The ICON® (CN; DMG-America, Ridgefield Park, NJ) resin infiltrant was used as a positive control in this study as it is a well-established commercial product known for its effectiveness in treating WSLs. This study hypothesized that BAG-based resin infiltrants would exhibit superior physicochemical and enhanced remineralization capabilities compared to conventional resin infiltrants. These infiltrants are anticipated to demonstrate improved performance under simulated oral conditions, making them a viable and effective treatment option for WSLs.

## Materials and methods

After obtaining ethical approval (IRB-2023-02-280) from the institutional review board at Imam Abdulrahman Bin Faisal University, Dammam, Saudi Arabia, the process of preparing and analyzing the samples commenced.

Formation of BAG-based resin infiltrants

The analytical grade chemicals utilized in this study were acquired (Sigma Aldrich in St. Louis, MO). The photoinitiators were ethyl 4-dimethylamine benzoate (EDAB) and camphorquinone (CQ), while the resin monomers were triethylene glycol dimethacrylate (TEGDMA) and diurethane dimethacrylate (UDMA). Following ratio optimization, a mixture consisting of 24.5% UDMA and 74.5% TEGDMA (converted to weight percentages) was prepared and left to stir at room temperature for 60 minutes. Following the addition of 0.5 wt. % of CQ and 0.5 wt. % of EDBA, the mixture was stirred for a further 60 minutes in a dark spot. The resulting material, referred to as the pure resin (PR), served as the negative control group. A commercial resin infiltrant (ICON) was used as a positive control group.

The synthesized resin infiltrants were blended with three different types of bioactive glasses, i.e., bioglass 45S5 (G018-144; Schott Glass AG, Mainz, Germany), fluoridated bioactive glass (F-BAG), and boron substituted bioactive glass (B-BAG) at a concentration of 2.5 wt. % in separate containers to construct the experimental resin infiltrants (optimization was done before finalization). First, the mixture was stirred by hand for 60 minutes. Next, it was stirred magnetically for 24 hours at 220 rpm in an open atmosphere. Subsequently, the blend was subjected to an ultrasonic shaker for 30 minutes. The RIS, RIB, and RIF were the names given to 45S5, B-BAG, and F-BAG-based resin infiltrants, respectively.

Sample size calculation

The samples were subjected to several analytical and characterization methods. The sample size was calculated using mean and standard deviation (SD) from a previously published study [20], The power calculation formula, with the ClinCalc software, means, and SD of the study was used with 80% and a confidence interval of 95%. The obtained sample size was five for each group with a significant level of p = 0.05.

Specimen preparation

Disc-shaped specimens (n = 5) for each group were prepared using a silicone mold (6 mm × 6 mm × 2 mm). The samples were cured using light curing equipment (3M ESPE Elipar S10 LED, 3M ESPE, St. Paul, MN) for 40 s. The matrix strip sheets have been used during light curing to produce a smooth sample surface. After curing, the resin discs were polished using a MetaServ250 polisher (BUEHLER, Lake Bluff, IL), progressing from medium grit (1500) to fine grit (2000) sandpaper. The process was completed with OptiDisc finishing discs (Kerr, Kloten, Switzerland) to achieve a smooth surface. The same procedure was applied to prepare the control groups.

Artificial saliva immersion

The chemicals used to manufacture the artificial saliva are mentioned in Table 1. Each disc specimen was immersed in an artificial saliva solution with a pH of 7 for thirty days. A portable pH meter (HI8424, Hanna Instruments, Inc., Woonsocket, RI) was utilized to continuously observe and ensure the stability of the solution’s pH levels. To maintain the efficacy of the solution, samples were dipped in the solution (10mL/sample as per the calculation of surface-to-volume ratio). All the samples were dipped in a glass container individually to maintain the maximum efficacy of the solution. The artificial saliva solution was replaced weekly to maintain its efficacy. On Day 0 and then on Day 30, the samples were evaluated for microhardness, surface roughness, and scanning electron microscopic analyses.

**Table 1:** Composition of artificial saliva [21,22]

Chemical name	Quantity (per 1000 mL)
NaCl	0.400 g
KCl	0.400 g
NaH_2_PO_4_. H_2_O	0.69 g
CaCl_2_. H_2_O	0.795 g
Na_2_S. 9H_2_O	0.005 g
NaOH	1 mM

pH cycling

The pH cycling procedure was performed based on previous research mentioned in the table. A new batch of disc samples was used to perform the pH cycling analysis. The composition of remineralizing and demineralizing solutions are mentioned in Table 2. The samples were immersed in a demineralizing solution for six hours, followed by an 18-hour immersion in a remineralizing solution. The incubator that housed the samples had a temperature setting of 37°C. For 30 days, the pH cycling process was carried out. The evaluations of microhardness, surface roughness, and morphology, were carried out on both Day 0 and Day 30 after the pH cycling.

**Table 2:** Composition of demineralization and remineralization solutions for pH cycling analysis [23]

Chemical	Demineralization solution	Remineralization solution
Name	Weight (g/1000 ml)	Weight (g/1000 ml)
CaCl_2_. H_2_O	0.244	0.166
NaH_2_PO_4_. H_2_O	0.36	0.15
CH₃COOH	3/1.42 ml	
KOH	Few drops to pH 4.4	
KCl		Few drops to pH 7.0

Vickers microhardness

The samples of each group were polished as mentioned earlier and the microhardness testing machine (MicroMet 6040 Microhardness Testing Machine, Buehler, Lake Bluff, IL) was used. During the test, a 200 g force was applied for a 20 s duration. The specified formula calculated the Vickers hardness number (HV).

HV = 1.8544(F/d2) 

Where F is the load (kgf), and d is the diagonal length (millimeters).

Surface roughness examination

Each sample's average three-dimensional surface roughness was measured using a contour GT surface roughness tester (Bruker ContourGT-K, Tucson, AZ). Three different measurements were taken for each specimen, and the average value was computed using both Day 0 and Day 30 treatment for artificial saliva and pH cycling analysis specimens. The surface roughness with its average served as the foundation for the outcomes.

Imaging analysis

After Day 30 immersion in artificial saliva and pH cycling solutions, the materials’ surface characteristic was examined with scanning electron microscopy (SEM, TESCAN VEGA-3 LMU, Brno, Czech Republic). To enhance imaging quality, a sputter coater was employed to apply a thin layer of gold coating to the samples for ninety seconds. Detailed images were captured using a magnification level of 2000x during the imaging process (where the voltage was 5-20 kV).

Statistical analysis

The data analysis was performed with SPSS Statistics software, version 20.0 (IBM Corp., Armonk, NY). When necessary, multiple groups were evaluated using one-way ANOVA or Kruskal-Wallis tests. The independent t-test and multiple comparison tests like Tukey or Mann-Whitney U were used. The Shapiro-Wilk test was employed to assess normality in the data, and p-values over 0.05 indicated that the distribution was normal.

## Results

Vickers microhardness - artificial saliva

Table 3 presents the results of the microhardness tests on disc samples on both Day 0 and Day 30 after immersion in artificial saliva. Initially, the CN group had the lowest microhardness value (42.19 ±4.15), and the RIF group had the highest (70.91 ±1.78). Significant differences were found within each group: RIS (p = 0.006), RIB (p = 0.001), RIF (p = 0.03), CN (p = 0.033), and PR (p = 0.006), on Day 30 of immersion, as well as between the groups (p = 0.001).

**Table 3:** The microhardness values of resin infiltrant (Day 0 and Day 30 after artificial saliva and pH cycling immersion) ^*^Shows a significant effect for Day 0 vs. Day 30 on microhardness within each group (p<0.05). Same alphabets ^a, b^ stand for insignificant differences

Vickers microhardness				
Groups	Day 0: artificial saliva, mean ±SD	Day 30: artificial saliva, mean ±SD	Day 0: pH cycling, mean ±SD	Day 30: pH cycling, mean ±SD
RIS	53.22 ±0.88^a^	67.46 ±0.81	57.02 ±1.98	54.92 ±1.09
RIB	50.93 ±1.04^a, b^	68.44 ±0.06	47.86 ±0.51^a^	46.13 ±0.65
RIF	70.91 ±1.78	78.20 ±0.06	69.32 ±2.64	64.15 ±1.89
CN	42.19 ±4.15	55.99 ±0.24	36.61 ±1.63	33.47 ±1.28
PR	48.58 ±1.90^b^	63.72 ±0.12	46.02 ±0.86^a^	43.84 ±0.46
P-value	0.001*	0.001*	0.001*	0.001*

On Day 30 of immersion, the RIF group still showed the highest microhardness value (78.20 ±0.06), while the CN group remained the lowest (55.99 ±0.24), with each group displaying statistically significant changes (p = 0.001).

Vickers microhardness - pH cycling

Table 3 presents the microhardness values of the disc samples after being subjected to pH cycling. On Day 0, RIF exhibited the highest microhardness (69.32 ±2.64), and CN showed the lowest (36.61 ±1.63). The differences in microhardness between RIS, RIB, RIF, CN, and PR were all statistically significant, with p-values of 0.003, 0.001, 0.002, 0.019, and 0.002, respectively. Significant differences were also noted within each group (p = 0.001).

After Day 30 in a pH cycling solution, RIF maintained the highest microhardness level (64.15 ±1.89), while CN remained the lowest (33.47 ±1.28), with these changes being statistically significant within the groups (p = 0.001).

Surface roughness - artificial saliva

**Figure 1:**
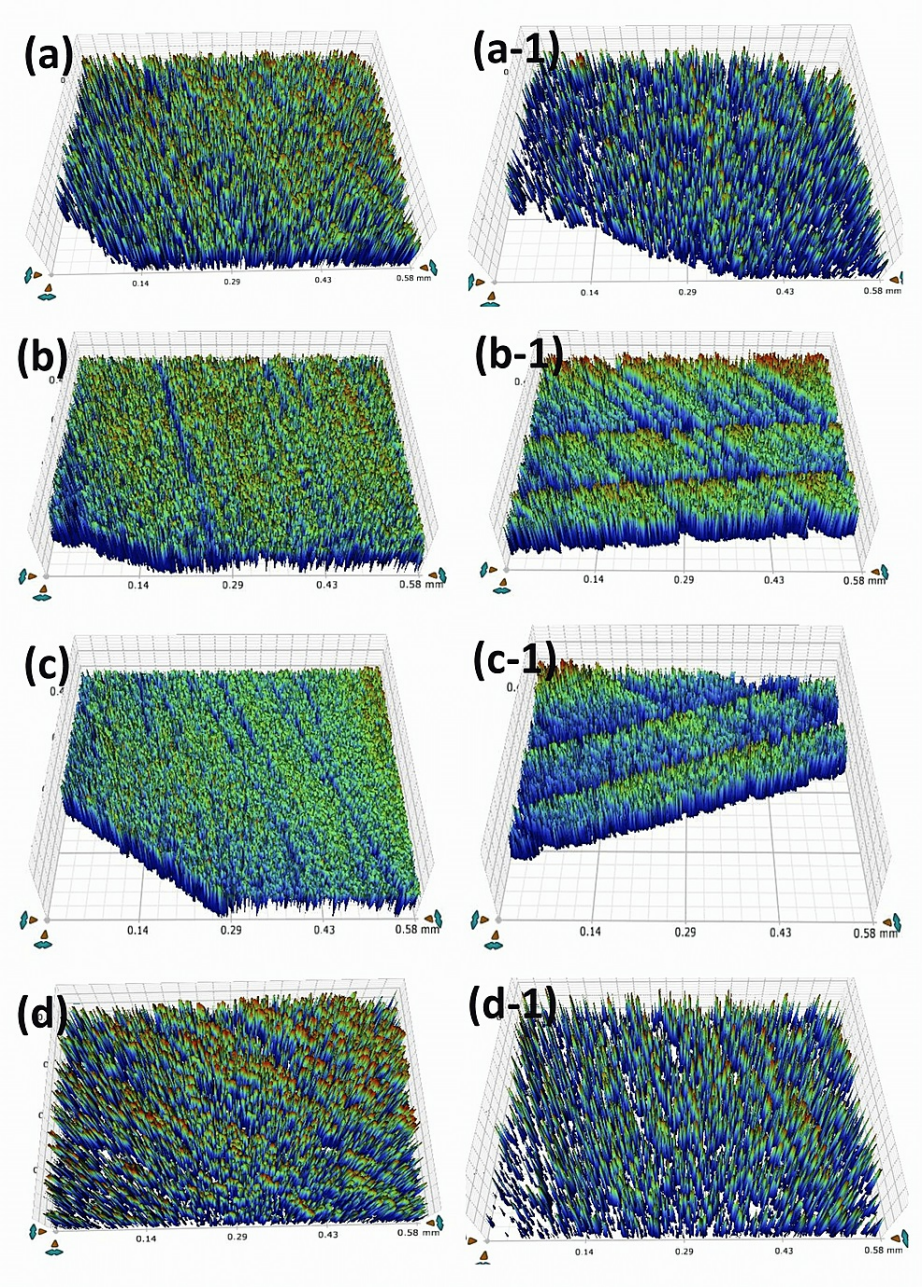
Images following the immersion into artificial saliva solution Day 0: the surface roughness view of disc samples of (a) RIS, (b) RIB, (c) RIF, and (d) CN groups. Day 30: the surface roughness view of disc samples of (a-1) RIS, (b-1) RIB, (c-1) RIF, and (d-1) CN groups

Figure 1 and Table 4 show the disc samples' surface roughness data on Day 0 and Day 30 after they were immersed in artificial saliva. The RIS group, on Day 0, had the lowest surface roughness value (0.58 ±0.03 µm), while the PR group had the highest (0.69 ±0.015 µm). On Day 0 of the immersion, there was no statistical significance in surface roughness between any of the groups. After Day 30, the differences between RIF and PR remained statistically insignificant; however, RIS (p = 0.004), RIB (p = 0.020), and CN (p = 0.040) showed significant differences.

**Table 4:** Analytic surface roughness values (µm) of groups (Day 0 and Day 30 of artificial saliva and pH cycling) *Shows significant effect for Day 0 vs. Day 30 on surface roughness within each group (p<0.05) Same alphabets a, b, c, d, e, f stand for significant differences

Surface roughness				
Groups	Day 0: artificial saliva, mean ±SD	Day 30: artificial saliva, mean ±SD	Day 0: pH cycling, mean ±SD	Day 30: pH cycling, mean ±SD
RIS	0.58 ±0.03^a, b, c^	1.09 ±0.05	0.57 ±0.04^a^	0.92 ±0.06
RIB	0.63 ±0.02^a, d^	1.14 ±0.001^a^	0.63 ±0.09^b^	0.92 ±0.33
RIF	0.62 ±0.007^b, e^	1.14 ±0.02	0.62 ±0.04^c^	0.94 ±0.54
CN	0.60 ±0.014^f^	1.07 ±0.06^a^	0.62 ±0.14^d^	0.91 ±0.63
PR	0.69 ±0.015^c, d, e, f^	1.12 ±0.03	0.80 ±0.05 ^a, b, c, d^	0.85 ±0.89
P-value	0.001*	0.026*	0.003*	0.304

Upon immersion in artificial saliva, the RIB resin group exhibited the highest measurement (1.14 ±0.001 µm) values, while the CN resin infiltrant showed the lowest (1.07 ±0.06 µm) values after Day 30. No significant statistical difference was found between the groups.

**Figure 2:**
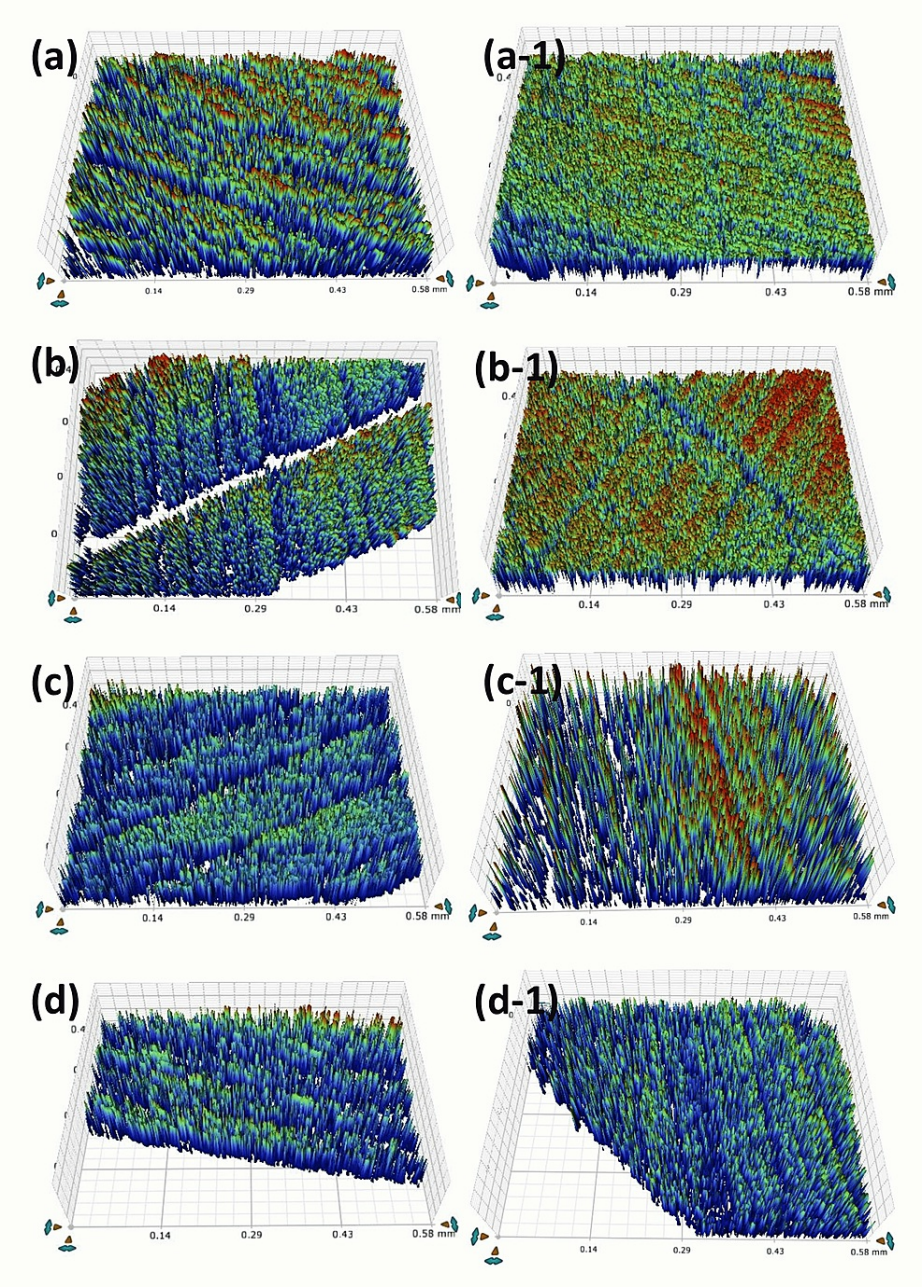
Images following the immersion into pH cycling solution Day 0: the surface roughness view of disc samples of (a) RIS, (b) RIB, (c) RIF, and (d) CN groups. Day 30: the surface roughness view of disc samples of (a-1) RIS, (b-1) RIB, (c-1) RIF, and (d-1) CN groups

Figure 2 and Table 4 display the surface roughness values for disc samples at Day 0 and Day 30 of pH cycling. On Day 0, the average roughness was highest (0.80 ±0.05 µm) in the PR group and lowest (0.57 ±0.04 µm) in the RIS group. Excluding the PR group, which showed no significant difference, all other resin groups showed significant differences when compared among themselves, with RIS, RIB, RIF, and CN having p-values of 0.0001, 0.004, 0.0001, and 0.032, respectively. Significant differences were also observed within each group (p = 0.005).

On day 30 of pH cycling, PR showed a decrease in roughness (0.85 ±0.89 µm), while RIF showed the highest roughness value (0.94 ±0.54 µm). However, these changes were not significant when comparing between groups.

SEM analysis - artificial saliva

**Figure 3:**
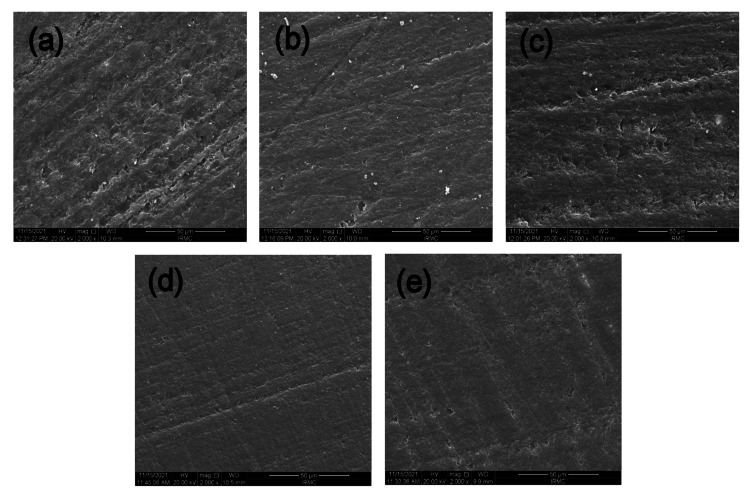
SEM micrographs post-immersion of resinous DISC samples into the artificial saliva solution (a) RIS resin, (b) RIB resin, (c) RIF resin (d) CN resin, and (e) PR resin. All images are magnified: x 2000 The SEM micrograph of the RIS resin after immersion in artificial saliva solution shows a relatively smooth surface with minimal porosity and a rougher surface. RIB shows a comparatively smoother surface to RIS, however roughness can still be observed. The RIF group appeared almost the same as the RIS surface; however, some obvious pitting can be noticed. CN group showed the smoothest surface among all the groups, and this could be due to the absence of any filler in its composition. The PR resin group is more or less showing the surface like the CN group, while still having some pitting on its surface

The results of 30 days of immersion in artificial saliva solution are shown in Figure 5. The RIS (Figure 3a) and RIF (Figure 3c) showed a rougher surface compared to the other experimental and commercial groups. RIB (Figure 3b), CN (Figure 3d), and PR (Figure 3e) appeared smoother than the other groups, along with some minor pits scattered on the surface. However, no material showed any prominent signs of cleavage or destruction of the surface.

SEM analysis - pH cycling solution

**Figure 4:**
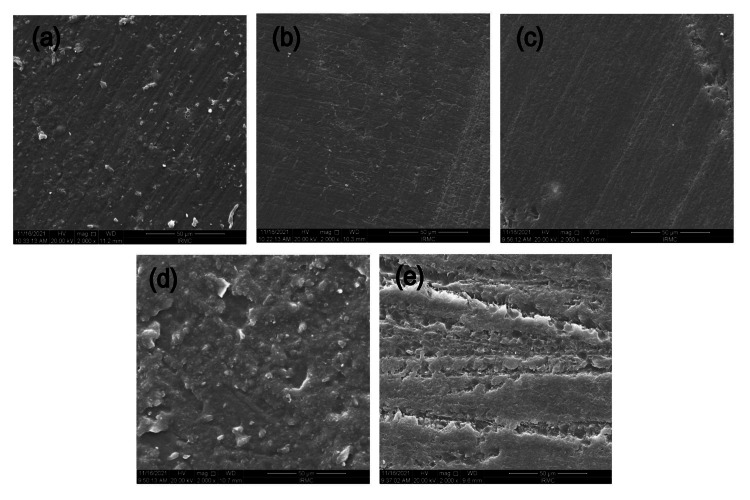
SEM micrographs post-immersion of resinous DISC samples into the pH cycling solution (a) RIS resin, (b) RIB resin, (c) RIF resin (d) CN resin, and (e) PR resin The SEM micrograph of the RIS resin after immersion in pH cycling solution shows some surface roughness with the filler particles scattered on the surface. The RIB showed a smoother surface without any filler particles or roughness. The RIF also showed a smooth surface without filler presence and or pitting. The CN exhibited a comparatively rougher surface than all other resins with the BAG fillers in them. The PR group also exhibited a rougher surface with obvious pitting

Figure 4 exhibits the effects of 30 days of immersion in a pH cycling solution. All experimental groups showed a substantial loss of surface smoothness, pitting, uneven surface texture, and dispersed filler particles, which were evident in the pH cycling condition. However, no cleavages or fissures were observed in any material sample. RIS (Figure 4a), CN (Figure 4d), and PR (Figure 4e) exhibited comparatively a rougher surface and the disappearance of the meshwork pattern, while CN and PR exhibited the development of small pits with rougher meshwork. RIS became visible, with several indications of filler particles dispersed across the surface. The RIB (Figure 4b) and RIF (Figure 4c) remained smoother with their surfaces with no signs of roughness, pitting, or cleavage.

## Discussion

BAG is incorporated with resin infiltrants to greatly increase their therapeutic potential in the treatment of early carious lesions and WSLs. When BAG particles come into contact with saliva, they release calcium and phosphate ions, which help the demineralized enamel remineralize. In addition to helping to halt the spread of carious lesions, this strengthens and repairs the tooth structure, something that standard resin infiltrants are unable to accomplish. The results of this study revealed that compared with the commercial resin infiltrant, the experimental doped with bioactive particles had superior chemical and mechanical properties. Following the artificial saliva immersion, all experimental resins and the commercial material [24] showed comparable increases in microhardness values. All the resins lost their microhardness values following pH cycling values, whereas the loss of microhardness values in all the experimental resins was comparatively less [25].

In the investigation of surface roughness, the RIS group demonstrated comparable surface roughness qualities of the material and demonstrated better resistance than all other experimental and commercial resin infiltrants [24]. The goals of this study were effectively met by analyzing the physicochemical characteristics of resins and adding three distinct kinds of BAG into them. Variations in the microhardness of direct restorative materials have a major impact on how long a resin-based dental repair lasts [26]. Following exposure to the experimental resin infiltrations, there was a noticeable increase in the microhardness values of every sample [27]. On the other hand, following the pH cycling trial, the greatest uniformity and the least relative decrease in microhardness were found. The initial hypothesis was dismissed due to statistically significant variations observed among the materials tested.

Following pH cycling treatment, RIB exhibited the lowest percentage changes among all the groups. The borate particles' nanostructure may be the cause of these modifications. The large surface area of the nanoparticles allows for a higher binding capability. Resilience against pH challenge was a result of the strong binding with the resin network [28]. The decrease of surface microhardness after Day 30 pH cycling may be caused by the hydrolytic breakdown of TEGDMA or by dissolving leftover minerals [29]. This drop in hardness is consistent with what Yazkan et al. [30] found. Incorporating bioactive glass particles into the infiltrants did not markedly alter the indentation site relative to the commercial material. Instead, it may have functioned as an ion reservoir and facilitated remineralization [31].

The samples were immersed in the solution for 30 days, and the microhardness values of the resin materials overall increased; however, when the samples were cycled through pH, the microhardness values decreased. The explanation might be that when the materials came into contact with the artificial saliva, the Ca and PO4 ions deposited on the disc's surfaces contributed to increasing their hardness [32]. Simultaneously, the acidic environment is anticipated to affect the material's characteristics, causing it to lose some of its constituent parts and hardness [33]. However, when immersed in artificial saliva, RIF demonstrated the highest levels of microhardness compared to the others. This supports the notion that fluoride alone may not be strong enough to resist acidic conditions. Yet, when fluoride combines with enamel hydroxyapatite, it forms fluorapatite, which is markedly more resistant [34]. The other BAG groups also exhibited promising results while evaluating the other groups. However, all the experimental groups outperformed the commercial resin material group, showing that experimental resins can perform better in clinical situations than commercial ones.

The study's findings showed that quick acid challenges produced the highest level of roughness, indicating that acid diffusion had broken down the resin-infiltrated barrier and exposed inadequately encapsulated enamel prisms. This is consistent with earlier studies that used the same demineralization and remineralization methods and showed that surface degradation occurred in response to acid attacks that occurred right away [35].

The experimental resin group's roughness escalated again after being subjected to pH cycling difficulties, eventually matching the CN group's similarly high level of roughness. This may result from changes in the polymeric chains of the invasive components brought on by encounters in an acidic environment [36]. However, research indicates that a gentler pH cycling method (involving a pH shift to 5.0 over 50 days) did not markedly change the microhardness of resin-infiltrated lesions following exposure to acid [30,37]. The presence of the hydrophilic monomer TEGDMA in the CN group rendered them vulnerable to absorbing water and potentially undergoing chemical degradation in an aqueous environment, leading to their solubility in water [38,39]. Since UDMA is more hydrophobic than TEGDMA, it was introduced to the experimental groups to address this problem.

Plaque retention and bacterial adherence are encouraged by surface roughness, which may shorten the restoration's lifespan. The mean ideal surface roughness values are 0.49-1.36 μm [40], which are clinically acceptable and work as an added benefit to the material's physical properties. All the experimental materials exhibited much lesser surface hardness values (even after Day 30 of immersion into the medium), thereby proving their efficacy and strong surface properties. The potential for the release of calcium, phosphorus, and fluoride ions, which can result in the formation of a new layer of apatite, is categorized as one of the glasses' bioactive characteristics and also plays a role in enhancing the rougher surface properties [41].

The appearance of the commercial material's roughness was also validated by some earlier research. In a profilometry investigation conducted by Taher et al. (2012) [42], resin infiltration with ICON revealed noticeably higher roughness values than fissure sealant. AFM and CT comparing ICON with fissure sealant and natural tooth further validated their findings [43]. Prior research has suggested that there was no statistically significant difference (p>0.05) in surface roughness values between the control group and the BAG-modified composites [44].

Given that the current research has been conducted only under laboratory conditions, further clinical studies are necessary to evaluate the long-term effectiveness of BAG-based resin infiltrant and its ability to restore minerals. With more research and clinical testing, which includes careful selection of cases and improvements to the composition to enhance the resin’s characteristics, BAG-based resins have the potential to be employed as a strategy for promoting remineralization, considering that laboratory conditions may vary from the dynamic and complex biological systems found in living organisms. In contrast, the authors of a different earlier investigation found that when the material was subjected to an acidic challenge, its polymeric chain began to chemically degrade and the material's roughness values rose [45].

The specimen surface on the side subjected to artificial saliva and pH cycling challenges had a higher surface roughness than the other surfaces in the representative SEM pictures of this investigation. The pictures, on the other hand, agreed with the numerical findings, which showed an increase in roughness [46]. The RIF group exhibited the highest microhardness values along with more resistance in terms of surface roughness, following artificial saliva and pH cycling challenges, and showed higher surface roughness values than others; it proves that the formation of apatite layer is more on RIF than enhances its microhardness. It also proves that the BAG-doped resin infiltrants can perform better than the commercial resin material in different physical and chemical challenges.

## Conclusions

Our findings revealed that BAG-doped resin infiltrants designed for research purposes could be more effective than the commercially available material in treating WSLs. The investigation found that these experimental materials performed better than the commercially available resin infiltrant, ICON®. After artificial saliva and pH cycling challenges, the experimental materials exhibited better physical and chemical properties. However further studies with larger sample sizes and clinical inclusion are required to validate these results.
